# Protective Effects Induced by Two Polyphenolic Liquid Complexes from Olive (*Olea europaea*, mainly *Cultivar Coratina*) Pressing Juice in Rat Isolated Tissues Challenged with LPS

**DOI:** 10.3390/molecules24163002

**Published:** 2019-08-19

**Authors:** Lucia Recinella, Annalisa Chiavaroli, Giustino Orlando, Luigi Menghini, Claudio Ferrante, Lorenzo Di Cesare Mannelli, Carla Ghelardini, Luigi Brunetti, Sheila Leone

**Affiliations:** 1 Department of Pharmacy, “G. d’Annunzio” University, 66013 Chieti, Italy; 2 Department of Neuroscience, Psychology, Drug Research and Child Health - NEUROFARBA - Pharmacology and Toxicology Section, University of Florence, 50139 Florence, Italy

**Keywords:** *Cultivar Coratina*, inflammation, oxidative stress, hydroxytyrosol

## Abstract

MOMAST^(®)^ HY100 and MOMAST^(®)^ HP30 are polyphenolic liquid complexes from olive pressing juice with a total polyphenolic content of 100 g/kg (at least 50% as hydroxytyrosol) and 36 g/kg (at least 30% as hydroxytyrosol), respectively. We investigated the potential protective role of MOMAST^(®)^ HY100 and MOMAST^(®)^ HP30 on isolated rat colon, liver, heart, and prefrontal cortex specimens treated with *Escherichia coli* lipopolysaccharide (LPS), a validated ex vivo model of inflammation, by measuring the production of prostaglandin (PG)E_2_, 8-iso-PGF_2α_, lactate dehydrogenase (LDH), as well as cyclooxygenase (COX)-2, tumor necrosis factor α (TNFα), and inducible nitric oxide synthase (iNOS) mRNA levels. MOMAST^(®)^ HY100 decreased LPS-stimulated PGE_2_ and LDH levels in all tested tissues. Following treatment with MOMAST^(®)^ HY100, we found a significant reduction in iNOS levels in prefrontal cortex and heart specimens, COX-2 and TNFα mRNA levels in heart specimens, and 8-iso-PGF_2α_ levels in liver specimens. On the other hand, MOMAST^(®)^ HP30 was found to blunt COX-2, TNFα, and iNOS mRNA levels, as well as 8-iso-PGF_2α_ in cortex, liver, and colon specimens. MOMAST^(®)^ HP30 was also found to decrease PGE_2_ levels in liver specimens, while it decreased iNOS mRNA, LDH, and 8-iso-PGF_2α_ levels in heart specimens. Both MOMAST^(®)^ HY100 and MOMAST^(®)^ HP30 exhibited protective effects on multiple inflammatory and oxidative stress pathways.

## 1. Introduction

It has been well established that olive tree (*Olea europaea*) polyphenols have healthy beneficial effects, including the prevention of several chronic diseases, such as cancer and aging-associated degenerative diseases [1,2]. These beneficial properties could be mainly related to the antioxidant activity of olive tree polyphenols, which were found able to both scavenge free radicals and reactive oxygen species and activate endogenous antioxidant enzymes, including glutathione peroxidase, glutathione reductase, and glutathione S-transferase [3,4,5]. Besides the antioxidant activity, anti-atherogenic, hepato-protective, hypoglycemic, anti-inflammatory, immunomodulatory, anticancer, and antimicrobic effects were also suggested for these compounds [5,6,7]. Hydroxytyrosol (HT) and the secoiridoid oleuropein (OE) are two abundant phenolic compounds in olives, virgin oil, and waste water from olive oil production [8,9,10]. Particularly, HT has antioxidant and scavenging activities comparable to oleuropein and catechol [11].

MOMAST^(®)^ HY100 and MOMAST^(®)^ HP30 (Bioenutra, Ginosa, TA, Italy) are polyphenolic liquid complexes from olive (*Olea europaea,* mainly *Cultivar Coratina*) pressing juice with a total polyphenolic content of 100 g/kg (at least 50% as HT) and 36 g/kg (at least 30% as HT), respectively (Table 1 and Table 2). In addition to HT, both MOMAST^(®)^ HY100 and MOMAST^(®)^ HP30 are also characterized by the presence of tyrosol and oleuropein.

Considering the antioxidant effects displayed by both HT and oleuropein, the aim of the present study was to investigate the putative protective effects of MOMAST^(®)^ HY100 and MOMAST^(®)^ HP30, both including HT and oleuropein, on the burden of oxidative stress/inflammation occurring on various isolated rat tissue (i.e., colon, liver, heart, and prefrontal cortex) specimens exposed to *Escherichia coli* lipopolysaccharide (LPS), a well-established inflammatory stimulus. Specifically, we studied the effects of MOMAST^(®)^ HY100 and MOMAST^(®)^ HP30 on multiple inflammatory and oxidative stress pathways, by measuring the production of prostaglandin (PG)E_2_, 8-iso-PGF_2α_, lactate dehydrogenase (LDH), as well as cyclooxygenase (COX)-2, tumor necrosis factor α (TNFα), and inducible nitric oxide synthase (iNOS) mRNA levels. The results support a rational use of these polyphenolic complexes in the prevention of tissue damage occurring during inflammation.

## 2. Results and Discussion

MOMAST^(®)^ HY100 (10, 50, and 100 µg/mL) and MOMAST^(®)^ HP30 (22, 110, and 220 µg/mL) were tested in vitro to evaluate their effects on cell viability. We observed that both polyphenolic liquid complexes were well tolerated by Hypo-E22 and C2C12 cell lines (Supplementary Figures S1–S2). Particularly, C2C12 and Hypo-E22 cell viability resulted in the limit of biocompatibility (>70 and <130% compared to the untreated control group) after exposition to polyphenolic extracts, in the respective tested concentration range, corresponding to identical concentrations of HT (5–50 µg/mL), which were in agreement with previous in vitro studies [11].

**Figure 1:**
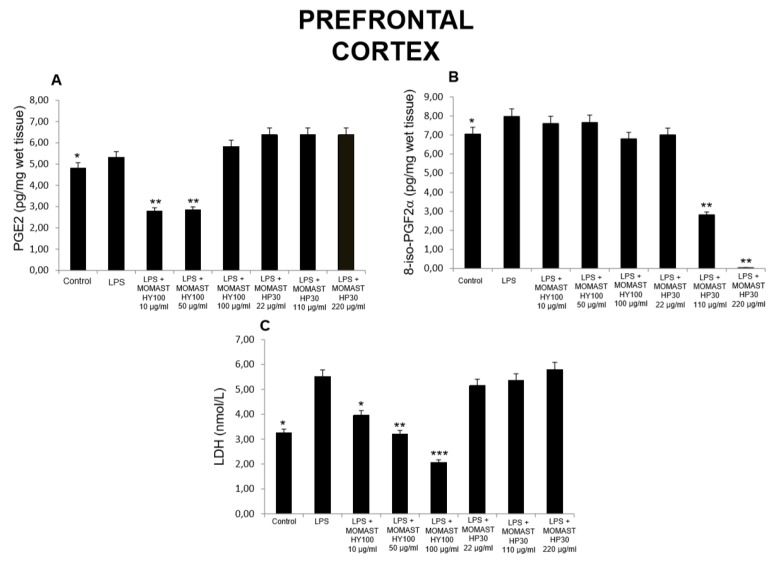
Effects of MOMAST(^®^) HY100 (10, 50, and 100 µg/mL) and MOMAST(^®^) HP30 (22, 110, and 220 µg/mL) on (**A**) PGE_2_ levels (pg/mg wet tissue), (**B**) 8-iso-prostaglandin F_2α_ (8-iso-PGF_2α_) levels, and (**C**) lactate dehydrogenase (LDH) activity (nmol/L) in rat prefrontal cortex specimens. Data were reported as means ± SEM. ANOVA, *p* < 0.01; *post-hoc* test, * *p* < 0.05, ** *p* < 0.01, *** *p < 0.001* vs. lipopolysaccharide (LPS)-treated group.

**Figure 2:**
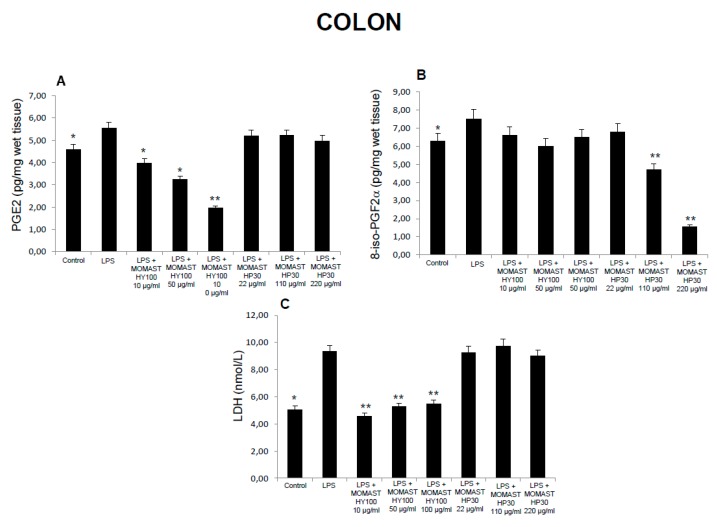
Effects of MOMAST(^®^) HY100 (10, 50, and 100 µg/mL) and MOMAST(^®^) HP30 (22, 110, and 220 µg/mL) on (**A**) PGE_2_ levels (pg/mg wet tissue), (**B**) 8-iso-prostaglandin F_2α_ (8-iso-PGF_2α_) levels, and (**C**) lactate dehydrogenase (LDH) activity (nmol/L) in colon specimens. Data were reported as means ± SEM. ANOVA, P < 0.01; *post-hoc* test, * *p* < 0.05, ** *p* < 0.01 vs. LPS-treated group.

**Figure 3:**
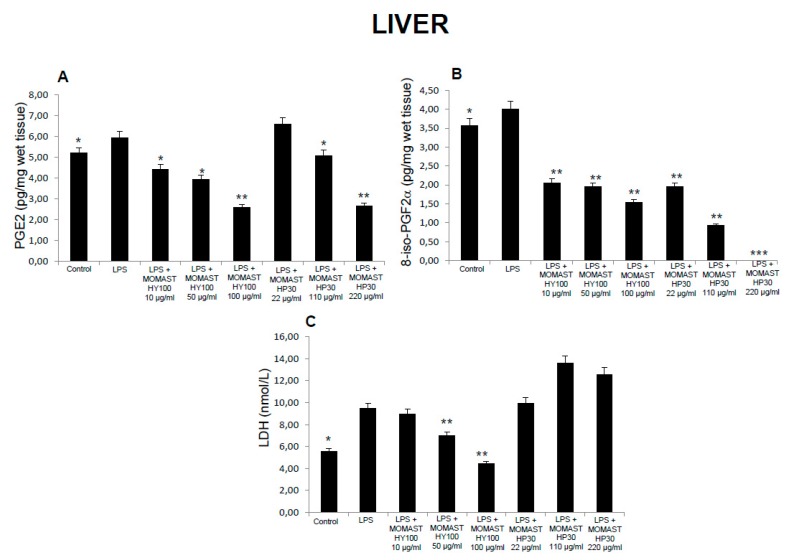
Effects of MOMAST(^®^) HY100 (10, 50, and 100 µg/mL) and MOMAST(^®^) HP30 (22, 110, and 220 µg/mL) on (**A**) PGE_2_ levels (pg/mg wet tissue), (**B**) 8-iso-prostaglandin F_2α_ (8-iso-PGF_2α_) levels, and (**C**) lactate dehydrogenase (LDH) activity (nmol/L) in rat liver specimens. Data were reported as means ± SEM. ANOVA, *p* < 0.01; *post-hoc* test, * *p* < 0.05, ** *p* < 0.01, *** *p* < 0.001 vs. LPS-treated group.

**Figure 4:**
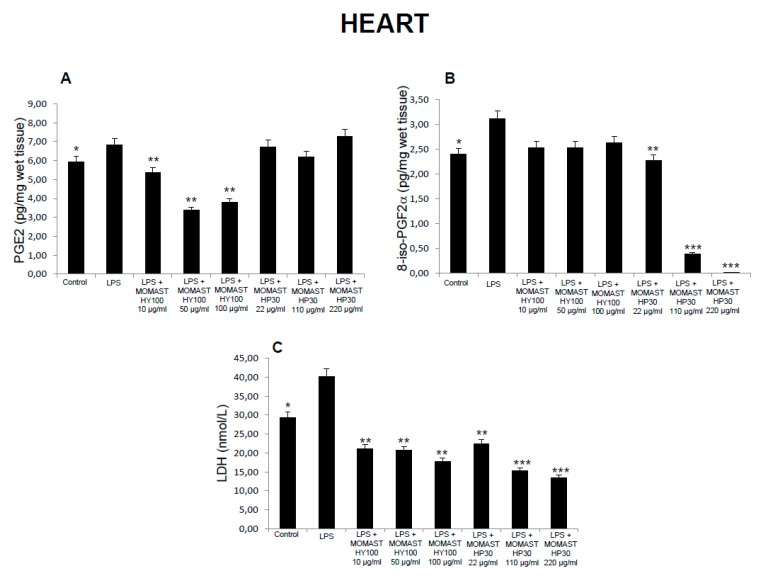
Effects of MOMAST(^®^) HY100 (10, 50, and 100 µg/mL) and MOMAST(^®^) HP30 (22, 110, and 220 µg/mL) on (**A**) PGE_2_ levels (pg/mg wet tissue), (**B**) 8-iso-prostaglandin F_2α_ (8-iso-PGF_2α_) levels, and (**C**) lactate dehydrogenase (LDH) activity (nmol/L) in rat heart specimens. Data were reported as means ± SEM. ANOVA, *p* < 0.01; *post-hoc* test, * *p* < 0.05, ** *p* < 0.01, *** *p* < 0.001 vs. LPS-treated group.

Considering these findings, we performed a second set of experiments aimed to evaluate the modulatory effects of MOMAST^(®)^ HY100 (10, 50, and 100 µg/mL) and MOMAST^(®)^ HP30 (22, 110, and 220 µg/mL) supplementation on oxidative stress and multiple inflammatory pathways in colon, liver, heart, and prefrontal cortex specimens challenged with LPS. As previously reported [12,13,14], isolated tissues challenged with LPS is a validated ex vivo experimental model to evaluate the modulatory effects of herbal extracts and drugs on inflammatory pathways and oxidative stress. The beneficial effects of plant polyphenols in humans have been confirmed by a large body of evidence [15,16,17,18]. A number of studies confirmed the antioxidant, anti-atherogenic, and protective effects of olive polyphenols, such as OLE and HT, against coronary artery disease [19,20,21,22,23]. In particular, HT, deacetoxy oleuropein aglycon, and oleuropein aglycon were classified as the strongest antioxidants in virgin olive oils [24]. Oxidative stress is defined as an imbalance in the pro-oxidant/antioxidant homeostasis, where increased production of reactive oxygen/nitrogen species (ROS/RNS) and free radicals can induce peroxidation reactions on biomolecules including proteins, lipids, and nucleic acids [25,26]. Oxidative damage is thought to play a key role in the pathogenesis of various chronic diseases, including cancer, atherosclerosis, cardiovascular diseases, chronic inflammation, and diabetes [27,28,29]. 8-Iso-PGF_2α_, an isomer of prostaglandins produced by free radical-catalyzed peroxidation of membrane arachidonic acid, is a stable marker of lipid peroxidation and oxidative stress [30]. We found that MOMAST^(®)^ HP30 (110 and 220 µg/mL) was able to decrease 8-iso-PGF_2α_ levels on rat prefrontal cortex, colon, liver, and heart tissues, challenged with LPS inflammatory stimulus (Figure 1, Figure 2, Figure 3 and Figure 4).

**Figure 5:**
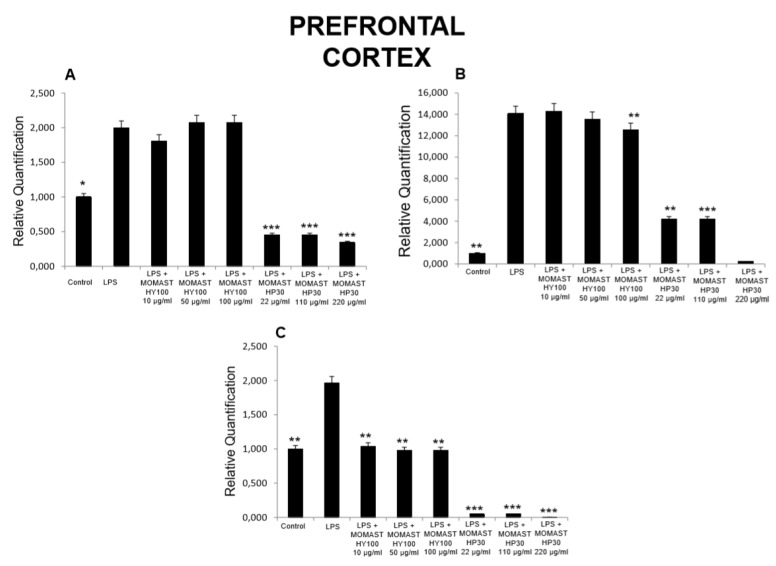
Effects of MOMAST(^®^) HY100 (10, 50, and 100 µg/mL) and MOMAST(^®^) HP30 (22, 110, and 220 µg/mL) on (**A**) cyclooxygenase (COX)-2, (**B**) tumor necrosis factor α (TNFα), and (**C**) inducible nitric oxide synthase (iNOS) in rat prefrontal cortex specimens. Data were reported as means ± SEM. ANOVA, *p* < 0.01; *post-hoc* test, * *p* < 0.05, ** *p* < 0.01, *** *p* < 0.001 vs. LPS-treated group.

**Figure 6:**
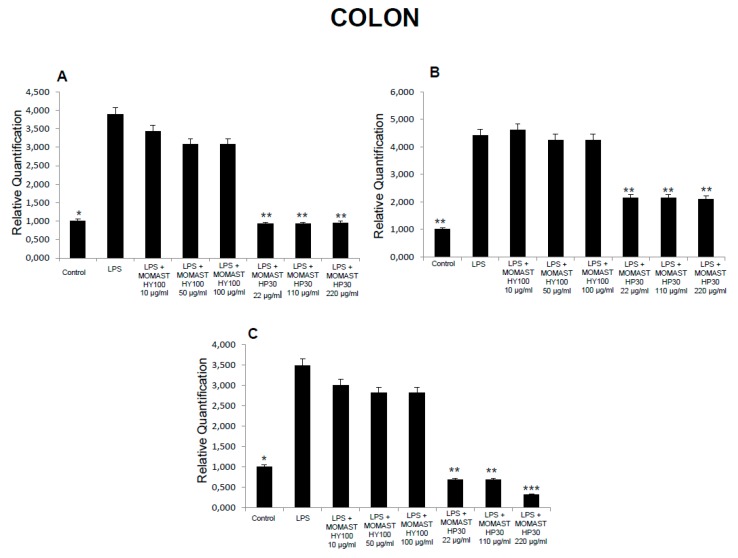
Effects of MOMAST(^®^) HY100 (10, 50, and 100 µg/mL) and MOMAST(^®^) HP30 (22, 110, and 220 µg/mL) on (**A**) cyclooxygenase (COX)-2, (**B**) tumor necrosis factor α (TNFα), and (**C**) inducible nitric oxide synthase (iNOS) in rat colon specimens. Data were reported as means ± SEM. ANOVA, *p* < 0.01; *post-hoc* test, * *p* < 0.05, ** *p* < 0.01, *** *p* < 0.001 vs. LPS-treated group.

**Figure 7:**
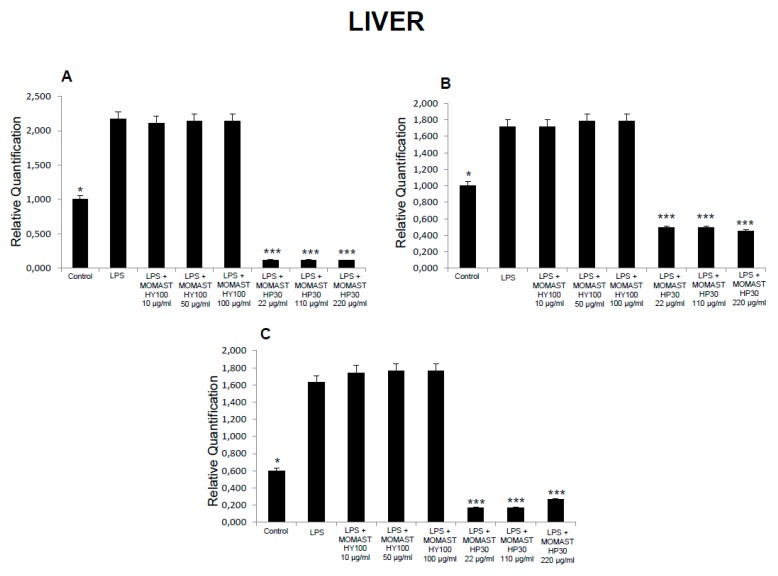
Effects of MOMAST(^®^) HY100 (10, 50, and 100 µg/mL) and MOMAST(^®^) HP30 (22, 110, and 220 µg/mL) on (**A**) cyclooxygenase (COX)-2, (**B**) tumor necrosis factor α (TNFα), and (**C**) inducible nitric oxide synthase (iNOS) in rat liver specimens. Data were reported as means ± SEM. ANOVA, *p* < 0.01; *post-hoc* test, * *p* < 0.05, *** *p* < 0.001 vs. LPS-treated group.

Moreover, MOMAST^(®)^ HY100 (10, 50, and 100 µg/mL) was effective in inhibiting LPS-induced 8-iso-PGF_2α_ in rat liver specimens (Figure 3B). These effects could be related, at least in part, to the free radical-reducing and -scavenging properties of HT [31,32], which is found in very high amounts in MOMAST^(®)^ HY100 and MOMAST^(®)^ HP30 (at least 50% and 30% of the phenolic fraction, respectively). HT was also shown to decrease low density lipoproteins oxidation [33], platelet aggregation [34], and 5- and 12-lipoxygenase activity [35] in vitro. However, we cannot exclude that our findings could also be related to other phenolic compounds which are present, even if in low content, in both liquid complexes. In this context, the antioxidant activity of OE has been widely confirmed both in vitro and in vivo [36]. We also investigated the activity of MOMAST^(®)^ HY100 and MOMAST^(®)^ HP30 on LDH level in inflamed tissues. LDH is a cytosolic enzyme, which can be considered a marker of tissue destruction [37,38]. Additionally, decreased LDH activity after treatment with herbal extracts has been related to protective effects in chronic inflammatory disorders such as inflammatory bowel disease (IBD) [39]. Following MOMAST^(®)^ HY100 (10, 50, and 100 µg/mL) treatment, we found a significant inhibition of LPS-induced LDH level in all tested tissues (Figure 1, Figure 2, Figure 3 and Figure 4). MOMAST^(®)^ HP30 (22, 110, and 220 µg/mL) was also able to decrease LDH level induced by LPS in heart specimens (Figure 7C). Actually, the reduction of LDH level could be related to the presence of HT [40] in both extracts, and further supports the protective effects induced by MOMAST^(®)^ HY100 and MOMAST^(®)^ HP30. Finally, we evaluated the modulatory effects of MOMAST^(®)^ HY100 and MOMAST^(®)^ HP30 on pro-inflammatory markers, including PGE_2_, COX-2, TNFα, and iNOS. LPS was found to induce macrophage production of inflammatory cytokines such as TNFα, interleukin-1β (IL-1β), and IL-6, along with inflammatory mediators including nitric oxide (NO) and PGE_2_ [41,42]. COX-2, an inducible enzyme stimulated by mitogenic and inflammatory stimuli, including LPS and cytokines, is known to be mainly involved in the synthesis of pro-inflammatory PGE_2_ in both neoplastic and inflamed tissues [43]. Similarly, iNOS, whose expression is induced by exposure to a number of stimuli, including LPS and TNFα, is involved in the generation of large amounts of NO, which plays a pivotal role in acute and chronic inflammation [44,45,46]. Following LPS inflammatory stimulus, we observed that MOMAST^(®)^ HP30 was able to reduce COX-2, TNFα, and iNOS mRNA levels in prefrontal cortex, colon, and liver specimens (Figure 5, Figure 6 and Figure 7).

**Figure 8:**
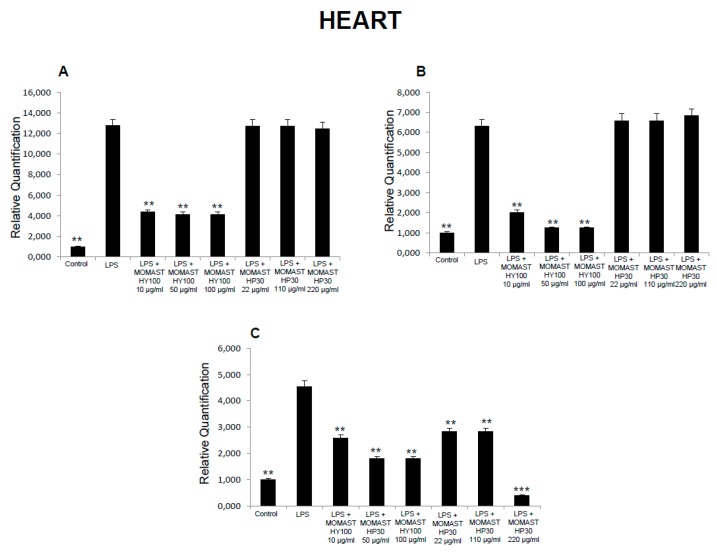
Effects of MOMAST(^®^) HY100 (10, 50, and 100 µg/mL) and MOMAST(^®^) HP30 (22, 110, and 220 µg/mL) on (**A**) cyclooxygenase (COX)-2, (**B**) tumor necrosis factor α (TNFα), (**C**) and inducible nitric oxide synthase (iNOS) in rat heart specimens. Data were reported as means ± SEM. ANOVA, *p* < 0.01; *post-hoc* test, ** *p* < 0.01, *** *p* < 0.001 vs. LPS-treated group.

On the other hand, COX-2, TNFα, and iNOS mRNA levels were decreased by MOMAST^(®)^ HY100 in heart tissue specimens (Figure 8). iNOS mRNA levels were also decreased by MOMAST^(®)^ HY100 in prefrontal cortex specimens (Figure 5C) and by MOMAST^(®)^ HP30 in heart tissue specimens (Figure 8C).

As regards PGE_2_, we found that while MOMAST^(®)^ HY100 was effective in reducing LPS-induced PGE_2_ levels in all tested tissues (Figure 1, Figure 2, Figure 3 and Figure 4), MOMAST^(®)^ HP30 was able to decrease PGE_2_ levels only in liver tissue (Figure 5A). The inhibitory effects induced by MOMAST^(®)^ HY100 and MOMAST^(®)^ HP30 on PGE_2_ levels, along with COX-2, TNFα, and iNOS mRNAs, support the protective effects of both polyphenolic liquid complexes in prefrontal cortex, colon, liver, and heart specimens. Accordingly, HT was shown to exert anti-inflammatory effects in LPS-stimulated RAW264.7 mouse macrophages by suppressing nuclear factor-*k*B (NF-κB) signaling and downregulating gene expression of iNOS, COX-2, TNFα, and IL-1β, and production of NO and PGE_2_ [47]. We hypothesize that the protective effects induced by MOMAST^(®)^ HY100 and MOMAST^(®)^ HP30 could also be related to the presence of other phenolic compounds, including tyrosol and OE. In this context, tyrosol was found to significantly inhibit COX-2 gene and protein expression, as well as PGE_2_ secretion in human glioblastoma cells [48]. Moreover, it has been found that OE significantly downregulated NO, COX-2, iNOS, and TNF-α in RAW264.7 macrophages following LPS treatment [49]. However, our findings indicate that MOMAST(^®^) HY100 and MOMAST(^®^) HP30 could display different effects in tissues. On one hand, Takeda et al. [50] reported that HT was able to suppress iNOS expression and NO production without any effect on NF-kB, COX-2, and TNFα expression in mouse peritoneal macrophages challenged with LPS; on the other hand, Maiuri et al. [51] and Zhang et al. [52] showed that HT was able to inhibit LPS-stimulated NFκB activation as well as COX-2 gene expression, in J774 murine macrophages and human monocytic THP-1 cells, respectively.

In conclusion, both MOMAST^(®)^ HY100 and MOMAST^(®)^ HP30 exhibited protective effects as indicated by the blunting effect on the tested pro-inflammatory mediators. On the basis of these results, HT seems to be the main extract component involved in the pharmacological effects. Nevertheless, considering the inherent limitations of the ex vivo experimental model, further investigations including oxidative stress and inflammation biomarkers in in vivo studies are needed for a more accurate evaluation of MOMAST^(®)^ HY100 and MOMAST^(®)^ HP30 efficacy.

## 3. Materials and Methods

### 3.1. In Vitro Studies

Rat Hypo-E22 cells (Cedarlane Cellution Biosystem) and mouse myoblast C2C12 cell lines (ATCC^®^ CRL-1772™) were cultured in Dulbecco’s modified eagle medium (DMEM) supplemented with 10% (*v*/*v*) heat-inactivated fetal bovine serum and 1.2% (*v*/*v*) penicillin G/streptomycin in 75 cm^2^ tissue culture flask (*n* = 5 individual culture flasks for each condition). The cultured cells were maintained in a humidified incubator with 5% CO_2_ at 37 °C. For cell differentiation, Hypo-E22 and C2C12 cell suspensions at a density of 1 × 106 cells/mL were treated with various concentrations (10, 50, and 100 ng/mL) of phorbol myristate acetate (PMA, Fluka) for 24 h or 48 h (induction phase). Thereafter, the PMA-treated cells were washed twice with ice-cold pH 7.4 phosphate buffer solution (PBS) to remove PMA and non-adherent cells, whereas the adherent cells were further maintained for 48 h (recovery phase). The morphology of the cells was examined under an inverted phase-contrast microscope. To assess the basal cytotoxicity of MOMAST^(®)^ HY100 and MOMAST^(®)^ HP30, a viability test was performed on 96-microwell plates, using a 3-(4,5-dimethylthiazol-2-yl)-2,5-diphenyltetrazolium bromide (MTT) test. Cells were incubated with MOMAST(^®^) HY100 (10, 50, and 100 µg/mL) and MOMAST(^®^) HP30 (22, 110, and 220 µg/mL), corresponding to HT (5, 25, and 50 µg/mL, respectively), for 24 h. Ten microliters of MTT (5 mg/mL) were added to each well and incubated for 3 h. The formazan dye formed was extracted with dimethyl sulfoxide and the absorbance was recorded as previously described [12,52]. The effects on cell viability were evaluated in comparison to the untreated control group.

### 3.2. Ex Vivo Studies

Male adult Sprague-Dawley rats (200–250 g) were housed in Plexiglass cages (40 cm × 25 cm × 15 cm), two rats per cage, in climatized colony rooms (22 ± 1 °C; 60% humidity), on a 12 h/12 h light/dark cycle (light phase: 07:00–19:00 h), with free access to tap water and food, 24 h/day throughout the study, with no fasting periods. Rats were fed a standard laboratory diet (3.5% fat, 63% carbohydrate, 14% protein, 19.5% other components without caloric value; 3.20 kcal/g).

Housing conditions and experimentation procedures were strictly in accordance with the European Union ethical regulations on the care of animals for scientific research.

According to the recognized ethical principles of “Replacement, Refinement, and Reduction of Animals in Research”, colon, liver, heart, and prefrontal cortex specimens were obtained as residual material from vehicle-treated rats randomized in our previous experiments approved by the Local Ethical Committee of University “G. d’Annunzio” and the Italian Health Ministry (Italian Health Ministry authorization N. 880, delivered on 24th August 2015). Rats were sacrificed by CO_2_ inhalation (100% CO_2_ at a flow rate of 20% of the chamber volume per min) and colon, liver, heart, and prefrontal cortex specimens were immediately collected and maintained in a humidified incubator with 5% CO_2_ at 37 °C for 4 h, in DMEM buffer with added bacterial LPS (10 μg/mL) (incubation period).

During the incubation period, tissues were treated with scalar concentrations of MOMAST(^®^) HY100 (10, 50, and 100 µg/mL) and MOMAST(^®^) HP30 (22, 110, and 220 µg/mL). Tissue supernatants were collected, and the PGE_2_ and 8-iso-PGF_2α_ levels (ng/mg wet tissue) were measured by radioimmunoassay (RIA), as previously reported [13,53]. Briefly, specific anti-8-iso-PGF_2α_ and anti-PGE_2_ were developed in the rabbit; the cross-reactivity against other prostanoids is <0.3%. One hundred microliters of prostaglandin standard or sample were incubated overnight at 4 °C with the 3H-prostaglandin (3000 cpm/tube; NEN) and antibody (final dilution: 1:120,000; kindly provided by Prof. G. Ciabattoni), in a volume of 1.5 mL of 0.025 M phosphate buffer. Free and antibody-bound prostaglandins were separated by the addition of 100 μL 5% bovine serum albumin and 100 μL 3% charcoal suspension, followed by centrifuging for 10 min at 4,000× *g* at 5 °C and decanting off the supernatants into scintillation fluid (Ultima Gold™, Perkin Elmer, Waltham, MA, USA) for β emission counting. The detection limit of the assay method was 0.6 pg/mL. Additionally, tissue supernatants were assayed for lactate dehydrogenase (LDH) activity [54]. LDH activity was measured by evaluating the consumption of nicotinamide adenine dinucleotide dehydrogenase (NADH) in 20 mM HEPES-K+ (pH 7.2), 0.05% bovine serum albumin, 20 μM NADH, and 2mM pyruvate using a microplate reader (excitation 340 nm, emission 460 nm) according to manufacturer′s protocol (Sigma-Aldrich, St. Louis, MO). LDH activity was measured by evaluating the consumption of NADH in 20 mM HEPES-K+ (pH 7.2), 0.05% bovine serum albumin, 20 μM NADH and 2 mM pyruvate using a microplate reader (excitation 340 nm, emission 460 nm) according to manufacturer′s protocol. In addition, individual prefrontal cortex, colon, liver, and heart specimens were quickly dissected to evaluate cyclooxygenase (COX)-2, tumor necrosis factor α (TNFα), and inducible nitric oxide synthase (iNOS) gene expression, as previously reported [55,56]. Tissue specimens were dissected and stored in RNAlater solution (Life Technologies, Carlsbad, CA, USA) at −20 °C until further processed. Total RNA was extracted from the tissues using TRI Reagent (Sigma-Aldrich, St. Louis, MO, USA) according to manufacturer’s protocol. One microgram of total RNA extracted from each sample in a 20-μL reaction volume was reverse transcribed using a high capacity cDNA reverse transcription kit (Life Technologies, Carlsbad, CA, USA). Reactions were incubated in a 2720 thermal cycler (Life Technologies, Carlsbad, CA, USA) initially at 25 °C for 10 min, then at 37 °C for 120 min, and finally at 85 °C for 5 s. Gene expression was determined by quantitative real-time PCR using TaqMan probe-based chemistry (Life Technologies, Carlsbad, CA, USA). Reactions were performed in MicroAmp Fast Optic 96-well Reaction Plates (Life Technologies, Carlsbad, CA, USA) on an ABI PRISM 7900 HT fast real-time PCR system (Life Technologies, Carlsbad, CA, USA). PCR primers and TaqMan probes were obtained from Life Technologies (Assays-on-Demand Gene Expression Products, Rn01483828_m1 for COX-2 gene, Rn01525859_g1 for TNFα, Rn00561646_m1 for iNOS. β-actin (Life Technologies, Carlsbad, CA, USA, Part No. 4352340E) was used as the housekeeping gene. The real-time PCR was carried out in triplicate. Data were elaborated with the sequence detection system (SDS) software version 2.3 (Applied Biosystems, Foster City, CA, USA). The comparative 2^−ΔΔCt^ method was used to quantify the relative abundance of mRNA and then determine the relative changes in individual gene expression (relative quantification) [57].

### 3.3. Statistical Analysis

Statistical analysis was performed using GraphPad Prism version 5.01 for Windows (GraphPad Software, San Diego, CA, USA). Means ± S.E.M. were determined for each experimental group and analyzed by one-way analysis of variance (ANOVA), followed by Newman–Keuls comparison multiple test. As for gene expression analysis, 1.00 (calibrator sample) was considered the theoretical mean for the comparison [57]. Statistical significance was set at *p* < 0.05. The number of animals randomized for each experimental group was calculated on the basis of the “Resource Equation” N = (E + T)/T (10 ≤ E ≤ 20) [58,59,60], according to the guidelines suggested by the National Centre for the Replacement, Refinement, and Reduction of Animals in Research (NC3RS) and reported on the following web site: https://www.nc3rs.org.uk/experimental-designstatistics.

## Figures and Tables

**Table 1:** Characteristics of the polyphenolic complex MOMAST^(®)^ HY 100.

Name:	MOMAST HY 100
Description:	Polyphenolic active complex of hydroxytyrosol—Liquid, with total polyphenolic content of 100 g/kg
Source Type:	Mainly *Cultivar Coratina*
Physical State:	Liquid
Appearance:	Light brown to brown liquid
Moisture:	N.A.
Ash:	Less than 10% (600 °C)
Total heavy (as Pb):	Less than 10 ppm
Total Plate Count:	Less than 100 cfu/g
Pesticides:	Absence
**Polyphenolic content measured through high performance liquid chromatography (HPLC)**	
Hydroxytyrosol (HPLC):	50 g/Kg
Tyrosol (HPLC)	15 g/kg
Oleuropein (HPLC)	0.5 g/Kg
Total Polyphenols (HPLC)	100 g/Kg

**Table 2:** Characteristics of the Polyphenolic Complex MOMAST^(®)^ HP 30.

Name:	MOMAST HP 30
Description:	Polyphenolic active complex from olives’ pressing juice—Liquid, with total polyphenolic content of 30 g/kg
Source Type:	Mainly *Cultivar Coratina*
Fisic State:	Liquid
Appearance:	Brown liquid
Moisture:	N. A.
Ash:	Less than 10% (600 °C)
Total heavy (as Pb):	Less than 10 ppm
Total Plate Count:	Less than 100 cfu/g
Pesticides:	Absence
Polyphenolic Content	
Hydroxytyrosol (HPLC):	15 g/Kg
Tyrosol (HPLC)	3 g/kg
Oleuropein (HPLC)	0.2 g/Kg
Total Polyphenols (HPLC)	30 g/Kg

## Supplementary Materials

The following are available on line: Supplementary Figure S1: Effects of MOMAST^(®)^ HY100 (10, 50, and 100 µg/mL) and MOMAST(^®^) HP30 (22, 110, and 220 µg/mL) on HypoE22 cell line viability. Supplementary Figure S2: Effects of MOMAST^(®)^ HY100 (10, 50, and 100 µg/mL) and MOMAST(^®^) HP30 (22, 110, and 220 µg/mL) on C2C12 cell line viability.

## References

[1] Carrera-González M., Ramírez-Expósito M., Mayas M., Martínez-Martos J. (2013). Protective role of oleuropein and its metabolite hydroxytyrosol on cancer. Trends Food Sci. Technol..

[2] Rahmani H.A., Albutti S.A., Aly M.S. (2014). Therapeutics role of olive fruits/oil in the prevention of diseases via modulation of anti-oxidant, anti-tumour and genetic activity. Int. J. Clin. Exp. Med..

[3] Masella R., Di Benedetto R., Varì R., Filesi C., Giovannini C. (2005). Novel mechanisms of natural antioxidant compounds in biological systems: involvement of glutathione and glutathione-related enzymes. J. Nutr. Biochem..

[4] Tundis R., Loizzo M.R., Menichini F., Statti G.A., Menichini F. (2008). Biological and pharmacological activities of iridoids: recent developments. Mini Rev. Med. Chem..

[5] Gorzynik-Debicka M., Przychodzen P., Cappello F., Kuban-Jankowska A., Marino Gammazza A., Knap N., Wozniak M., Gorska-Ponikowska M. (2018). Potential Health Benefits of Olive Oil and Plant Polyphenols. Int. J. Mol. Sci..

[6] Tripoli E., Giammanco M., Tabacchi G., Di Majo D., Giammanco S., La Guardia M. (2005). The phenolic compounds of olive oil: structure, biological activity and beneficial effects on human health. Nutr. Res. Rev..

[7] Fabiani R., de Bartolomeo A., Rosignoli P., Servili M., Selvaggini R., Montedoro G.F., di Saverio C., Morozzi G. (2006). Virgin olive oil phenols inhibit proliferation of human promyelocytic leukemia cells (HL60) by inducing apoptosis and differentiation. J. Nutr..

[8] Angerosa F., d’Alessandro N., Corana F., Mellerio G. (1996). Characterization of phenolic and secoiridoid aglycons present in virgin olive oil by gas chromatography-chemical ionization mass spectrometry. J. Chromatogr..

[9] Cinquanta L., Esti M., La Notte E. (1997). Evolution of phenolic compounds in virgin olive oil during storage. J. Am. Oil Chem. Soc..

[10] El S.N., Karakaya S. (2009). Olive tree (Olea europaea) leaves: Potential beneficial effects on human health. Nutr. Rev..

[11] Martínez L., Ros G., Nieto G. (2018). Hydroxytyrosol: Health Benefits and Use as Functional Ingredient in Meat. Medicines (Basel).

[12] Ferrante C., Recinella L., Ronci M., Menghini L., Brunetti L., Chiavaroli A., Leone S., Di Iorio L., Carradori S., Tirillini B. (2019). Multiple pharmacognostic characterization on hemp commercial cultivars: Focus on inflorescence water extract activity. Food Chem. Toxicol..

[13] Locatelli M., Macchione N., Ferrante C., Chiavaroli A., Recinella L., Carradori S., Zengin G., Cesa S., Leporini L., Leone S. (2018). Graminex Pollen: Phenolic Pattern, Colorimetric Analysis and Protective Effects in Immortalized Prostate Cells (PC3) and Rat Prostate Challenged with LPS. Molecules.

[14] Mollica A., Stefanucci A., Zengin G., Locatelli M., Macedonio G., Orlando G., Ferrante C., Menghini L., Recinella L., Leone S. (2018). Polyphenolic composition, enzyme inhibitory effects ex-vivo and in-vivo studies on two Brassicaceae of north-central Italy. Biomed. Pharmacother..

[15] Covas M.I., Nyyssönen K., Poulsen H.E., Kaikkonen J., Zunft H.J., Kiesewetter H., Gaddi A., de la Torre R., Mursu J., Bäumler H. (2006). The effect of polyphenols in olive oil on heart disease risk factors: A randomized trial. Ann. Intern. Med..

[16] Camargo A., Ruano J., Fernandez J.M., Parnell L.D., Jimenez A., Santos-Gonzalez M., Marin C., Perez-Martinez P., Uceda M., Lopez-Miranda J. (2010). Gene expression changes in mononuclear cells in patients with metabolic syndrome after acute intake of phenol-rich virgin olive oil. BMC Genomics.

[17] de Bock M., Derraik J.G., Brennan C.M., Biggs J.B., Morgan P.E., Hodgkinson S.C., Hofman P.L., Cutfield W.S. (2013). Olive (Olea europaea L.) leaf polyphenols improve insulin sensitivity in middle-aged overweight men: a randomized, placebo-controlled, crossover trial. PLoS ONE.

[18] Medina-Remón A., Tresserra-Rimbau A., Pons A., Tur J.A., Martorell M., Ros E., Buil-Cosiales P., Sacanella E., Covas M.I., Corella D. (2015). Effects of total dietary polyphenols on plasma nitric oxide and blood pressure in a high cardiovascular risk cohort. The PREDIMED randomized trial. Nutr. Metab. Cardiovasc. Dis..

[19] Malik N.S., Bradford J.M. (2006). Changes in oleuropein levels during differentiation and development of floral buds in ‘Arbequina’olives. Sci. Horticult..

[20] Manna C., D’Angelo S., Migliardi V., Loffredi E., Mazzoni O., Morrica P., Galletti P., Zappia V. (2002). Protective effect of the phenolic fraction from virgin olive oils against oxidative stress in human cells. J. Agric. Food Chem..

[21] Visioli F., Bellosta S., Galli C. (1998). Oleuropein, the bitter principle of olives, enhances nitric oxide production by mouse macrophages. Life Sci..

[22] Carluccio M.A., Siculella L., Ancora M.A., Massaro M., Scoditti E., Storelli C., Visioli F., Distante A., de Caterina R. (2003). Olive oil and red wine antioxidant polyphenols inhibit endothelial activation. Arterioscler. Thromb. Vasc. Biol..

[23] Edgecombe S.C., Stretch G.L., Hayball P.J. (2000). Oleuropein, an antioxidant polyphenol from olive oil, is poorly absorbed from isolated perfused rat intestine. J. Nutr..

[24] Carrasco-Pancorbo A., Cerretani L., Bendini A., Segura-Carretero A., Del Carlo M., Gallina-Toschi T., Lercker G., Compagnone D., Fernández-Gutiérrez A. (2005). Evaluation of the antioxidant capacity of individual phenolic compounds in virgin olive oil. J. Agric. Food Chem..

[25] Uttara B., Singh A.V., Zamboni P., Mahajan R.T. (2009). Oxidative stress and neurodegenerative diseases: A review of upstream and downstream antioxidant therapeutic options. Curr. Neuropharmacol..

[26] Halliwell B., Whiteman M. (2004). Measuring reactive species and oxidative damage in vivo and in cell culture: How should you do it and what do the results mean?. Br. J. Pharmacol..

[27] Fridovich I. (1999). Fundamental aspects of reactive oxygen species, or what’s the matter with oxygen?. Ann. N. Y. Acad. Sci..

[28] Fang Y.Z., Yang S., Wu G. (2002). Free radicals, antioxidants, and nutrition. Nutrition.

[29] Matsuda M., Shimomura I. (2013). Increased oxidative stress in obesity: Implications for metabolic syndrome, diabetes, hypertension, dyslipidemia, atherosclerosis, and cancer. Obes. Res. Clin. Pract..

[30] Praticò D., Lee V.M.Y., Trojanoswki J.Q., Rokach J., FitzGerald G.A. (1998). Increased F2-isoprostanes in Alzheimer’s disease: evidence for enhanced lipid peroxidation in vivo. FASEB J..

[31] Mateos R., Madrona A., Pereira-Caro G., Domínguez V., Cert R.M., Parrado J., Sarriá B., Bravo L., Espartero J.L. (2015). Synthesis and antioxidant evaluation of isochroman-derivatives of hydroxytyrosol: Structure-activity relationship. Food Chem..

[32] Jemai H., El Feki A., Sayadi S. (2009). Antidiabetic and antioxidant effects of hydroxytyrosol and oleuropein from olive leaves in alloxan-diabetic rats. J. Agric. Food Chem..

[33] Salami M., Galli C., De Angelis L., Visioli F. (1995). Formation of F2-isoprostanes in oxidized low density lipoprotein: inhibitory effect of hydroxytyrosol. Pharmacol. Res..

[34] Petroni A., Blasevich M., Salami M., Papini N., Montedoro G.F., Galli C. (1995). Inhibition of platelet aggregation and eicosanoid production by phenolic components of olive oil. Thromb. Res..

[35] Kohyama N., Nagata T., Fujimoto S., Sekiya K. (1997). Inhibition of arachidonate lipoxygenase activities by 2-(3,4-dihydroxyphenyl)ethanol, a phenolic compound from olives. Biosci. Biotechnol. Biochem..

[36] Speroni E., Guerra M.C., Minghetti A., Crespi-Perellino N., Pasini P., Piazza F., Roda A. (1998). Oleuropein evaluated in vitro and in vivo as an antioxidant. Phytother. Res..

[37] Manna S., Bhattacharyya D., Basak D.K., Mandal T.K. (2004). Single oral dose toxicity study of a-cypermethrin in rats. Indian J. Pharmacol..

[38] Nagarjun S., Dhadde S.B., Veerapur V.P., Thippeswamy B.S., Chandakavathe B.N. (2017). Ameliorative effect of chromium-d-phenylalanine complex on indomethacin-induced inflammatory bowel disease in rats. Biomed. Pharmacother..

[39] Kannan N., Guruvayoorappan C. (2013). Protective effect of Bauhinia tomentosa on acetic acid induced ulcerative colitis by regulating antioxidant and inflammatory mediators. Int. Immunopharmacol..

[40] Cabrerizo S., De La Cruz J.P., López-Villodres J.A., Muñoz-Marín J., Guerrero A., Reyes J.J., Labajos M.T., González-Correa J.A. (2013). Role of the inhibition of oxidative stress and inflammatory mediators in the neuroprotective effects of hydroxytyrosol in rat brain slices subjected to hypoxia reoxygenation. J. Nutr. Biochem..

[41] Lee J.D., Kato K., Tobias P.S., Kirkland T.N., Ulevitch R.J. (1992). Transfection of CD14 into 70Z/3 cells dramatically enhances the sensitivity to complexes of lipopolysaccharide (LPS) and LPS binding protein. J. Exp. Med..

[42] Yun K.J., Kim J.Y., Kim J.B., Lee K.W., Jeong S.Y., Park H.J., Jung H.J., Cho Y.W., Yun K., Lee K.T. (2008). Inhibition of LPS-induced NO and PGE2 production by asiatic acid via NF-kappa B inactivation in RAW 264.7 macrophages: possible involvement of the IKK and MAPK pathways. Int. Immunopharmacol..

[43] Subbaramaiah K., Dannenberg A.J. (2003). Cyclooxygenase 2: A molecular target for cancer prevention and treatment. Trends Pharmacol. Sci..

[44] Salvemini D., Ischiropoulos H., Cuzzocrea S. (2003). Roles of nitric oxide and superoxide in inflammation. Methods Mol. Biol..

[45] Denlinger L.C., Fisette P.L., Garis K.A., Kwon G., Vazquez-Torres A., Simon A.D., Nguyen B., Proctor R.A., Bertics P.J., Corbett J.A. (1996). Regulation of inducible nitric oxide synthase expression by macrophage purinoreceptors and calcium. J. Biol. Chem..

[46] Weisz A., Cicatiello L., Esumi H. (1996). Regulation of the mouse inducible-type nitric oxide synthase gene promoter by interferon-gamma, bacterial lipopolysaccharide and NG-monomethyl-L-arginine. Biochem. J..

[47] Yonezawa Y., Miyashita T., Nejishima H., Takeda Y., Imai K., Ogawa H. (2018). Anti-inflammatory effects of olive-derived hydroxytyrosol on lipopolysaccharide-induced inflammation in RAW264.7 cells. J. Vet. Med. Sci..

[48] Lamy S., Ben Saad A., Zgheib A., Annabi B. (2016). Olive oil compounds inhibit the paracrine regulation of TNF-α-induced endothelial cell migration through reduced glioblastoma cell cyclooxygenase-2 expression. J. Nutr. Biochem..

[49] Mao X., Xia B., Zheng M., Zhou Z. (2019). Assessment of the anti-inflammatory, analgesic and sedative effects of oleuropein from Olea europaea L.. Cell. Mol. Biol. (Noisy-le-grand)..

[50] Takeda Y., Bui V.N., Iwasaki K., Kobayashi T., Ogawa H., Imai K. (2014). Influence of olive-derived hydroxytyrosol on the toll-like receptor 4-dependent inflammatory response of mouse peritoneal macrophages. Biochem. Biophys. Res. Commun..

[51] Maiuri M.C., De Stefano D., Di Meglio P., Irace C., Savarese M., Sacchi R., Cinelli M.P., Carnuccio R. (2005). Hydroxytyrosol, a phenolic compound from virgin olive oil, prevents macrophage activation. Naunyn Schmiedebergs Arch. Pharmacol..

[52] Zhang X., Cao J., Jiang L., Zhong L. (2009). Suppressive effects of hydroxytyrosol on oxidative stress and nuclear Factor-kappaB activation in THP-1 cells. Biol. Pharm. Bull..

[53] Ferrante C., Recinella L., Locatelli M., Guglielmi P., Secci D., Leporini L., Chiavaroli A., Leone S., Martinotti S., Brunetti L. (2017). Protective Effects Induced by Microwave-Assisted Aqueous Harpagophytum Extract on Rat Cortex Synaptosomes Challenged with Amyloid β-Peptide. Phytother Res..

[54] Chiavaroli A., Recinella L., Ferrante C., Locatelli M., Carradori S., Macchione N., Zengin G., Leporini L., Leone S., Martinotti S. (2017). Crocus sativus, Serenoa repens and Pinus massoniana extracts modulate inflammatory response in isolated rat prostate challenged with LPS. J. Biol. Regul. Homeost. Agents.

[55] Menghini L., Leporini L., Vecchiotti G., Locatelli M., Carradori S., Ferrante C., Zengin G., Recinella L., Chiavaroli A., Leone S. (2018). stigmas and byproducts: Qualitative fingerprint, antioxidant potentials and enzyme inhibitory activities. Food Res. Int..

[56] Ferrante C., Orlando G., Recinella L., Leone S., Chiavaroli A., Di Nisio C., Shohreh R., Manippa F., Ricciuti A., Vacca M. (2016). Central apelin-13 administration modulates hypothalamic control of feeding. J. Biol. Regul. Homeost. Agents.

[57] Leone S., Chiavaroli A., Shohreh R., Ferrante C., Ricciuti A., Manippa F., Recinella L., Di Nisio C., Orlando G., Salvatori R. (2015). Increased locomotor and thermogenic activity in mice with targeted ablation of the GHRH gene. Growth Horm. IGF Res..

[58] Livak K.J., Schmittgen T.D. (2001). Analysis of relative gene expression data using real-time quantitative PCR and the 2(-Delta Delta C(T)). Methods.

[59] Charan J., Kantharia N.D. (2013). How to calculate sample size in animal studies?. J. Pharmacol. Pharmacother..

[60] Recinella L., Leone S., Ferrante C., Chiavaroli A., Shohreh R., Di Nisio C., Vacca M., Orlando G., Salvatori R., Brunetti L. (2017). Effects of growth hormone-releasing hormone gene targeted ablation on ghrelin-induced feeding. Growth Horm. IGF Res..

