# Supplementary material for: Protective Effects Induced by Two Polyphenolic Liquid Complexes from Olive (Olea europaea, mainly Cultivar Coratina) Pressing Juice in Rat Isolated Tissues Challenged with LPS
Source: Molecules. 2019 Aug 19;24(16):3002. doi: 10.3390/molecules24163002 (PMC6720671; doi:10.3390/molecules24163002)
Supplement: Supplementary file 1 [file molecules-24-03002-s001.pdf]

molecules

MDPI

Article

Protective effects induced by two polyphenolic liquid complexes from olive (*Olea europaea*, mainly *Cultivar Coratina*) pressing juice in rat isolated tissues challenged with LPS

Lucia Recinella[1,†], Annalisa Chiavaroli[1,†], Giustino Orlando [1], Luigi Menghini [1], Claudio Ferrante [1], Lorenzo Di Cesare Mannelli [2], Carla Ghelardini [2], Luigi Brunetti [1,*] and Sheila Leone [1].

[1] Department of Pharmacy, "G. d'Annunzio" University, 66013 Chieti, Italy
[2] Department of Neuroscience, Psychology, Drug Research and Child Health - NEUROFARBA - Pharmacology and Toxicology Section, University of Florence, 50139 Florence, Italy
* Correspondence: luigi.brunetti@unich.it; Tel.: +39 0871 3554758
† The two authors contributed equally to the work.

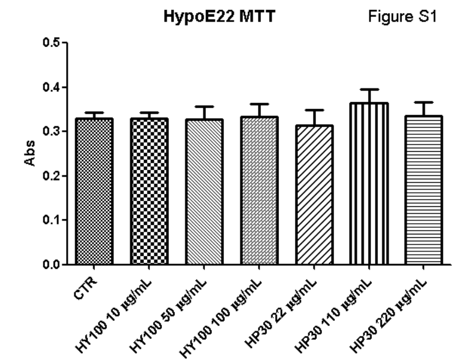

Figure S1. Effects of MOMAST(®) HY100 (10, 50, and 100 μg/mL) and MOMAST(®) HP30 (22, 110, and 220 μg/mL) on HypoE22 cell line viability.

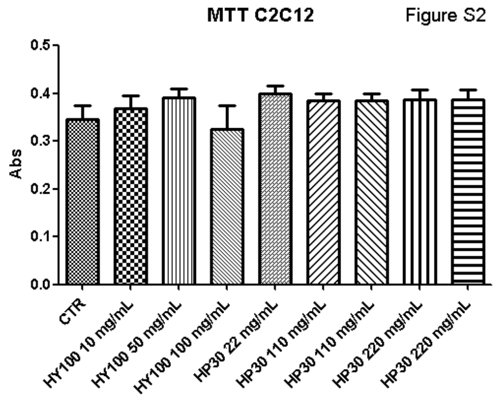

Figure S2. Effects of MOMAST(®) HY100 (10, 50, and 100 μg/mL) and MOMAST(®) HP30 (22, 110, and 220 μg/mL) on C2C12 cell line viability.
